# Health financing policies for aging populations: a comparative study of seven countries

**DOI:** 10.1186/s12913-025-13648-y

**Published:** 2025-11-26

**Authors:** Bashir Azimi Nayebi, Rouhollah Yaghoubi, Behzad Najafi, Rahim Khodayari-Zarnaq

**Affiliations:** 1 Department of Health Policy and Management, School of Management and Medical Informatics, Tabriz University of Medical Sciences, Tabriz, Iran; 2 Department of Health Management and Economics, Faculty of Health, Tehran University of Medical Sciences, Tehran, Iran; 3 Department of Health Economics, School of Management & Medical Informatics, Tabriz University of Medical Sciences, Tabriz, Iran; 4 Tabriz Health Services Management Research Centre, Health Management and Safety Promotion Research Institute, Tabriz University of Medical Sciences, Tabriz, Iran

**Keywords:** Health policy, Aging, Seniors, Healthcare financing, Financial support, Universal health coverage

## Abstract

**Background:**

Global population aging presents unprecedented challenges for healthcare systems, particularly in developing nations where demographic shifts strain existing infrastructure. This study investigates health financing mechanisms for older adult populations across seven representative countries (Argentina, Cuba, Russia, Romania, Thailand, Ukraine, and Sri Lanka) to identify policy solutions for sustainable geriatric care.

**Methods:**

Employing Arksey and Malley’s scoping review framework, we systematically analyzed peer-reviewed literature from five major databases (Ovid, PubMed, Web of Science, Scopus, ProQuest) and grey sources. Our methodology focused on three key financing models: government-subsidized programs, insurance schemes, and out-of-pocket expenditure systems.

**Results:**

Comparative analysis revealed that all seven nations have established distinct older adult healthcare financing approaches. A universal consensus emerged regarding the necessity of: age-specific policy frameworks, guaranteed service accessibility, and financial risk protection mechanisms. Notably, each country demonstrated heightened policy attention proportional to their aging population growth rates.

**Conclusion:**

The final review found that in the policies developed and implemented in the seven countries: age-specific policies, access to services, and financial risk protection mechanisms were consistently emphasized as the most important features. However, there were significant variations in implementation approaches, which were broadly categorized into three models: tax-based systems, social health insurance, and hybrid models.

**Supplementary information:**

The online version contains supplementary material available at 10.1186/s12913-025-13648-y.

## Background

Human society’s success in advancing medical science, improving people’s health and improving lifestyles has brought the world to the threshold of old age. Experts define old age as 60 years and older [1]. According to the World Health Organization, the proportion of older adult persons in the global population is expected to almost double from 12%, or 900 million persons, to 22%, or 2 billion persons, in the period 2015–2050. Although population ageing began in high-income countries, the fastest demographic transition now takes place in low- and middle-income countries [2]. In 2050, around 80% of older people will live in low- and middle-income countries, and countries like Iran, Chile, China and Russia will be in the same position that Japan finds itself in today [3].

While increasing life expectancy is an achievement and a worthy goal, it does not necessarily mean that older people are living healthy and active lives. There is little evidence that this increase in life expectancy is always accompanied by good health [2]. The growth of the older population and the increasing burden of chronic disease will be one of the greatest challenges to health systems in most countries of the world in the twenty-first century. Non-communicable diseases (NCDs) and the disability they cause in older people are a major part of the global burden of disease, and this burden is increasing. Older people, especially those in more disadvantaged socio-economic conditions, experience multiple types of disability and disease together [4–6]. Most health problems of older people can be prevented or delayed with timely and appropriate interventions. By creating integrated health systems and appropriate supportive environments, it is possible to ensure a dignified and quality life for older people [7].

The foregoing highlights the urgency of reforming and redesigning health systems to meet the needs of the older adult. Such reforms are essential for achieving the United Nations Sustainable Development Goals, particularly those related to health and well-being [8, 9]. The World Health Organization recommends that health and social care should be integrated and coherent to improve the functioning of older people and prevent the decline of their inherent capacities. Integrated care refers to the management and provision of services that promote, prevent, diagnose and treat health problems, as well as rehabilitation and palliative care, at different levels within and outside the health system, so that people are able to access the services they need at any time of life. Integrated care strategies should include different levels of service provision, including clinical (micro), organisational (meso) and systemic (macro) levels [7, 10]. Integrated care has been presented internationally as a policy solution. It is necessary to achieve the goals of sustainable development and universal health coverage(UHC), taking into account the ever-increasing growth of the world’s older adult population [11]. A healthy and long-lived society requires a multi-sectoral approach and the involvement and interaction of different sectors at different levels. Achieving the desired goals will require cooperation between governmental and non-governmental actors, including governmental, quasi-governmental, private and voluntary sector providers, academia and older people themselves. Therefore, a key step in strengthening policies is to create a coalition and a common understanding of the issue of older people, which in turn can enable multi-sectoral engagement [2, 12].

For this reason, this study has attempted to examine the policies developed in selected countries in the field of ageing in order to understand the World Health Organisation’s perspective of “a world with long and healthy lives for all”: A commitment to healthy ageing; the development of age-friendly environments; the adaptation of health care systems to the needs of older people; the development of stable and equitable long-term care (LTC) systems; and the improvement of information systems, measurement, monitoring and evaluation. the improvement of information systems, measurement, monitoring and research on healthy ageing and the involvement of older people in all relevant decision-making processes [13].

Accordingly, this study specifically addresses the following research questions:

- Did countries where the older adult population exceeds 10% have explicit financing plans for health services? If so, have these plans been successful in achieving their intended goals?
- What were the key similarities among these countries in designing their older adult care financing plans?
- Was financing for older adult services structured as a separate system, or was it integrated into the countries’ broader health and social welfare frameworks?Did these countries develop holistic, long-term strategies for addressing the needs of their aging populations, beyond just healthcare financing?

Therefore, this study has attempted to specifically examine and compare health financing policies and mechanisms for ageing populations in a selection of countries, in order to understand the different models and key policy components that have been implemented.

## Methods

In this study, the Arksey and O’Malley framework was used to conduct a scoping review on 1 November 2024. This framework consists of six steps: identifying the research question, identifying relevant studies, selecting and screening studies, categorizing data, summarizing and reporting results, and providing practical recommendations. Additionally, the PRISMA flowchart was used to design and implement the study. The checklist includes 20 essential reporting items and two optional ones, outlining the scope of the review [14].

### Identifying the research question

The main research question is: “What are the characteristics and effectiveness of financing policies for elderly healthcare services in developing countries?” This question is further broken down into the following sub-questions:

- How are financial resources for the elderly collected?
- How are these financial resources pooled?
- Finally, how are healthcare services for the elderly purchased?

### Identifying relevant studies

Relevant information was collected from the following databases: Ovid, PubMed, Web of Science, Scopus, ProQuest, and Google Scholar (Appendix 1 – Search Strategy).

The search keywords included: “(aging or aged or adult or older or elderly) and (”health insurance” or “health program” or “health programs” or “health financing” or “healthcare financing” or “health expenditure” or “healthcare expenditure” or “health cost” or “health costs” or “healthcare cost” or “healthcare costs” or “financial support”) and (Romania or Ukraine or Cuba or Russia or Thailand or Argentina or China or Sri Lanka)”. Additional sources used to identify relevant articles included:

- Manual searches in high-impact, relevant journals,
- Reviewing references of existing studies,
- Examining citations of included studies using Google Scholar,
- Consulting experts and professionals,
- Checking the websites of the World Health Organization (WHO) and other international organizations
- Checking the Ministry of Health websites of the countries under study

To identify grey literature, we searched the Health Care Management Information Consortium (HMIC), System for Information on Grey Literature in Europe (SIGLE), and the European Association for Grey Literature Exploitation (EAGLE).

***Inclusion Criteria:***

1. Studies addressing **purchasing of healthcare services** for older adults,
2. Studies examining mechanisms of **resource collection** for health financing,
3. Studies examining mechanisms of **resource pooling**,
4. Studies analyzing **health financing policies** in developing countries.

***Exclusion Criteria:***

- Studies that focus on the absence of health financing mechanisms?
- Studies that do not specifically assess health financing?
- Abstracts or conference proceedings without full-text availability
- Articles that did not provide sufficient information on the subject

### Selection and screening of studies

Selected articles were thoroughly reviewed, and relevant data were extracted using a structured data extraction form. The Endnote X21 reference management software was used for organizing sources, reviewing titles and abstracts, and identifying duplicates. The screening process was conducted in three stages: Title Screening: Articles were initially filtered based on their titles. Abstract Screening: Abstracts were reviewed, and irrelevant or conflicting studies were excluded. Full-Text Review: The full text of the remaining articles was analyzed, and data were manually extracted using content analysis methods.

Two researchers independently performed the data coding. The PRISMA flowchart was used to document the study selection and screening process.

### Categorization of data

A manual data extraction form was designed in Microsoft Word 2010. To refine the form, data from five randomly selected articles were extracted as a pilot test. Two researchers independently extracted data, and any ambiguities were resolved through discussions with the research team. The extracted data included: Author and year of publication, country where the study was conducted, study objective, type of Health system financing examined, strategies, revenue raising, pooling of funds, and purchasing of services.

### Summarizing and reporting results

Extracted data were manually analyzed using content analysis, a widely used method for identifying, analyzing, and reporting themes in qualitative data. [15, 16]The data analysis and coding process followed these steps:

- Familiarization with the content of the selected articles
- Identification and extraction of key themes
- Categorization of studies based on identified themes
- Reviewing and finalizing the results for each category
- Ensuring the reliability and consistency of the findings

### Providing guidance and practical recommendations

Based on the extracted results and input from the research team, recommendations and guidelines were developed. These findings were further discussed and structured into practical recommendations.

## Results

Table 1 shows the demographic information for Argentina, Cuba, Romania, Russia, Thailand, Ukraine and Sri Lanka. Russia has the highest number of older adult people and Cuba the lowest, while Romania has the highest percentage relative to population.

**Table 1:** Demographic information of selected countries

Country(Continent)	Population (Million People)(2021)^*^	Older adult over 65	Older adult percentage**	life expectancy***	Health system financing
Argentina(South America)	44.78	5,034,774	11.24	77.98	Social health insurance (65%) and the private sector (35%)
Cuba(North America)	11.33	1,764,062	15.57	78.33	General taxation (100%)
Romania(Europe)	19.36	3,638,608	18.79	75.14	Social Health Insurance (65%), State and Local Authorities Budget (15.45%), private sources (19.55%)
Russia(Europe, Asia)	145.8	22,018,759	15.09	74.57	Budgets (50%), Compulsory Medical Insurance Funds (30%), voluntary medical insurance (10%) and paid medical services (10%)
Thailand(Asia)	69.62	8,637,966	12.41	79.91	General taxes (50%), social health insurance contributions (30%), private insurance premiums (10%), and a low level of direct out-of-pocket payments (10%)
Ukraine(Europe)	43.99	7,349,111	16.70	73.02	State budget (100%)
Sri Lanka(South Asia)	21.32	2,311,283	10.84	76.80	General taxation (90%) and out-of-pocket (10%)

* The first factor is the population of more than ten million

** The second factor is the older adult population above ten percent

*** Worldometers(2024)

Beyond country-specific descriptions, the results were synthesized into a comparative framework based on three financing dimensions: (1) revenue raising, (2) pooling of resources, and (3) purchasing of services. This framework highlights structural similarities (e.g., reliance on tax-based financing in Cuba, Ukraine, Sri Lanka) and differences (e.g., Romania’s three-pillar pension and insurance model). Importantly, equity concerns such as high out-of-pocket costs in Sri Lanka and Ukraine emerged as critical cross-country issues.

**Fig. 1:**
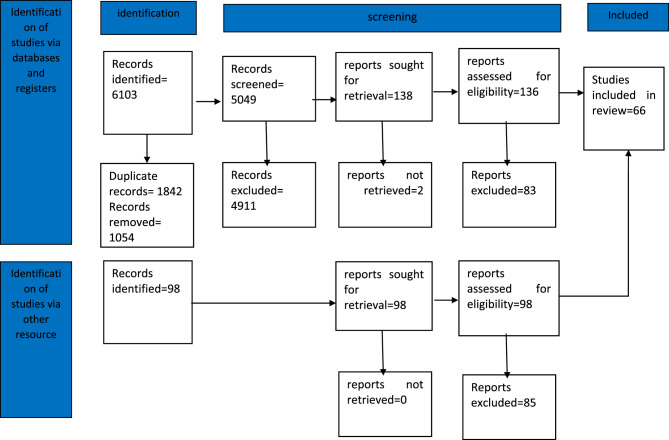
Prisma 2020 flow diagram for database and other source searches

Out of the 6,201 articles retrieved from the databases, 1,054 were removed due to duplication. In the title and abstract screening phase, 5,147 articles were reviewed. Of these, 4,911 were excluded based on title screening, and 168 were excluded based on abstract screening. Finally, the full text of 68 articles was assessed, from which 66 were selected after excluding studies due to lack of access, publication in other languages, insufficient information, or irrelevance (Fig. 1). were included after screening, and the analysis revealed three dominant models of older adult health financing: (a) tax-based systems (Cuba, Ukraine, Sri Lanka), (b) social health insurance (Argentina, Romania, Russia), and (c) hybrid systems (Thailand).

Overall, 66 studies (Table 2) were included after screening, and the analysis revealed three dominant models of older adult health financing: (a) tax-based systems (Cuba, Ukraine, Sri Lanka), (b) social health insurance (Argentina, Romania, Russia), and (c) hybrid systems (Thailand).

**Table 2:** Studies included in review

N	Title	Author/years	Setting/Types of studies	Country
1	Closing the Health Gaps for the Elderly in Thailand [17]	World Bank Group/2022	-/Report	Thailand
2	Voluntary private health insurance, health-related behaviours and health outcomes: evidence from Russia [18]	Andrey Aistov and … /2021	moral hazard/Longitudinal Monitoring Survey	Russia
3	Financial Risk Protection and Unmet Healthcare Need in Russia [19]	Zlatko Nikoloski, Jane Cheatley, Elias Mossialos/2021	financial risk protection/Longitudinal Monitoring Survey	Russia
4	Sustainability and Resilience in the Russian Health System [20]	Elena Aksenova and … /2021	international cooperation/analytical report	Russia
5	Long-Term Care Model: Review of Population Ageing Practices and Policies [21]	Duangjai Lorthanavanich Osuke Komazawa/2021	Long-term Care/Review	Thailand
6	Implementing health financing policies to overhaul the healthcare delivery system in Ukraine [22]	Carlos Avila/2021	Health system reform/Review Article	Ukraine
7	Sri Lanka Health System Review [23]	Lalini Rajapaksa and … /2021	Health system/review	Sri Lanka
8	Conditions for the Growth of the “Silver Economy” in the Context of Sustainable Development Goals: Peculiarities of Russia [24]	L. Reshetnikova; N. Boldyreva; M. Perevalova; S. Kalayda; Z. Pisarenko/2021	silver economy/multivariate statistical analysis	Russia
9	Analysis of Composition Change of Public Facility Care Users After the Universal Coverage Scheme in Thailand [25]	N. Meemon; S. C. Paek/2020	Thailand’s Universal Coverage Scheme/logistic regression	Thailand
10	Improving social protection in Romania [26]	Dragoș Adăscăliţei Cristina Raţ Marcel Spătari/2020	SOCIAL PROTECTION/Report	Romania
11	ROMANIA Country case study on the integrated delivery of long-term care [27]	WHO Regional/2020	long-term care/Report	Romania
12	Country Diagnostic Study on Long-Term Care in Thailand [28]	Asian Development Bank (ADB)/2020	Health system development/Report	Thailand
13	Estimating Demand for Long-term Care Insurance in Thailand: Evidence from a Discrete Choice Experiment [29]	W. Chandoevwit; N. Wasi/2019	long-term care insurance/	Thailand
14	Universal health coverage and primary care, Thailand [30]	K. Sumriddetchkajorn and … /2019	universal health coverage/Policy & Practice	Thailand
15	Managing Thailand’s Ageing Population [31]	Kwanchit Sasiwongsaroj and Youngyut Burasit/2019	Ageing Population/perspective	Thailand
16	UKRAINE REVIEW OF HEALTH FINANCING REFORMS 2016–2019 [32]	WHO–World Bank Joint Report/2019	financing reforms/Report	Ukraine
17	The Social Health Insurance Scheme for Pubic Sector Employees in Sri Lanka and Its Effect on Reducing the Financial Burden of Illness [33]	S. Karunaratna; T. Ranasinghe; N. Chandraratne; A. De Silva/2019	Financial Burden/cross-sectional	Sri Lanka
18	Accelerating reforms of primary health care towards universal health coverage in Sri Lanka [34]	S. Perera; and … /2019	universal health coverage/Report	Sri Lanka
19	GROWING OLD BEFORE BECOMING RICH CHALLENGES OF AN AGING POPULATION IN SRI LANKA [35]	ASIAN DEVELOPMENT BANK/2019	Ageing Population/Report	Sri Lanka
20	Inpatient care expenditure of the elderly with chronic diseases who use public health insurance: Disparity in their last year of life [36]	W. Chandoevwit; P. Phatchana/2018	Inpatient care expenditures/Cohort	Thailand
21	Economic considerations on Cuba public health and its relationship with universal health [37]	Ana María Gálvez González and … /2018	Universal Health Coverage/Report	Cuba
22	Ageing Related Policies and Priorities in the Implementation of the 2030 Agenda for Sustainable Development [38]	As reported in the Voluntary National Reviews/2018	Policies and Priorities/Agenda	Romania
23	Estimating Long-Term Care Costs among Thai Elderly: A Phichit Province Case Study [39]	Pattaraporn Khongboon and … /2018	Cost of LTC/logistic regression	Thailand
24	Health Care in Ukraine [40]	Orange Health Consultants/2018	Health Care/Report	Ukraine
25	Have out-of-pocket health care payments risen under free health care policy? The case of Sri Lanka [41]	A. Pallegedara; M. Grimm/2018	OOP/regression	Sri Lanka
26	Sri Lanka - Achieving pro-poor universal health coverage without health financing reforms [42]	Owen Smith/2018	high-performing health system/case study	Sri Lanka
27	Fiscal Policy for a Sustainable Healthcare and Pension System in Thailand under an Aging Population: Case Study from Japan [43]	Supanun Chumjai/2017	Pension System/Case Study	Thailand
28	Erratum to: Mortality and treatment costs of hospitalized chronic kidney disease patients between the three major health insurance schemes in Thailand [44]	S. Anutrakulchai and … /2016	Treatment costs/logistic regression	Thailand
29	The health care sector in EBRD countries of operations [45]	Pavel Dvorak and … /2016	health care sector/Report	Romania
30	Health Care & Long-Term Care Systems [46]	Country Documents/2016	health care/Report	Romania
31	Romania Health system review [47]	Cristian Vlaˇdescu and … /2016	health care/Report	Romania
32	Aging in Russia [48]	Olga Strizhitskaya/2016	retirement system/Review	Russia
33	Knowledge and Attitude toward the Selection of Health Insurance Type after Retirement in Ratchaburi Province, Thailand [49]	Supitcha Sumretphol, Prathurng Hongsranagon/2016	health insurance/cross-sectional survey	Thailand
34	Primary healthcare policy implementation in South Asia [50]	C. van Weel and … /2016	Primary healthcare/Case Study	Sri Lanka
35	The development of universal health insurance coverage in Thailand: Challenges of population aging and informal economy [51]	M. C. Hsu; X. G. Huang; S. Yupho/2015	Health insurance coverage/Review	Thailand
36	Ukraine: health system review [52]	V. Lekhan and … /2015	Health system/Review	Ukraine
37	Durable Financing of the Romanian Healthcare System within the European Background [53]	Valentin Antohi, Gabriel Cojocaru/2015	Financial resources/Review	Romania
38	The Kingdom of Thailand Health System Review [54]	Pongpisut Jongudomsuk and … /2015	Health System/Review	Thailand
39	Ukraine Health system review [55]	Valery Lekhan and … /2020	Health System/Review	Ukraine
40	Aging in Romania: research and public policy [56]	S. Bodogai; S. Cutler/2014	Aging/Policy	Romania
41	Healthcare use and voluntary health insurance after retirement in Thailand [57]	P. Kananurak/2014	Retirement/original research	Thailand
42	Retirement mutual aid associations – actors in social inclusion [58]	G. Stănilă/2014	Associations/case study	Romania
43	The Romanian Healthcare System and Financing Strategies [59]	Celia Dana Besciu/2014	Financing health care/case study	Romania
44	National Health Reform Strategy for Ukraine 2015–2020 [60]	Minister of Health of Ukraine/2014	Reform/Main Policy Issues	Ukraine
45	Social protection systems in Latin America and the Caribbean: Cuba [61]	Carmelo Mesa-Lago/2013	Social protection systems/Report	Cuba
46	Public health services, an essential determinant of health during crisis. Lessons from Cuba, 1989–2000 [62]	Pol De Vos and … /2012	Public health services/Retrospective study	Cuba
47	Explanation of inequality in utilization of ambulatory care before and after universal health insurance in Thailand [63]	V. Yiengprugsawan and … /2011	Universal Coverage Scheme/Original research	Thailand
48	Geriatric care and gerontological research in Argentina [64]	J. R. Jauregui and … /2011	Geriatric care/Review	Argentina
49	Supply vs. demand of health services in the actual demographic context of Romania [65]	Carmen Lavinia PANAIT/2011	Supply vs. Demand/Observational studies	Romania
50	Russian Federation Health system review [66]	Larisa Popovich and … /2011	Health System/Review	Russia
51	long-term care in Romania [67]	DANIELA POPA, MD/2010	LTC/Report	Romania
52	Primary Care in Cuba [68]	Stephanie Hauge/2007	Primary Care/Report	Cuba
53	Health financing in Argentina: an empirical study of health care expenditure and utilization: innovations in health financing [69]	Eleonora Cavagnero and … /2006	Health financing/Empirical study	Argentina
54	Health Financing in Argentina: An Empirical Study of Health Care Expenditure and Utilization [70]	Eleonora Cavagnero and … /2006	Health financing/Empirical study	Argentina
55	Healthcare rationing: a guide to policy directions in Sri Lanka [71]	N. Withanachchi; Y. Uchida/2006	Public health system/Guide	Sri Lanka
56	Comparison of health care financing in Egypt and Cuba: lessons for health reform in Egypt [72]	C.A. Gericke/2005	Health system/Review	Cuba
57	Health care for older persons in Argentina: A country profile [73]	M. Montero-Odasso and … /2004	Healthcare systems/International Health Affairs	Argentina
58	Knowledge-based changes to health systems: the Thai experience in policy development [74]	V. Tangcharoensathien and … /2004	Health systems/Original research	Thailand
59	Financing health services for pensioners in Argentina: A salutary tale [75]	P. Lloyd-Sherlock/2003	finance health care/Original research	Argentina
60	Comparison of four health systems: Cuba, China, Japan and the USA, an approach to reality [76]	Susana M Borroto Gutiérrez and … /2003	health systems/Review	Cuba
61	Health care reform in Russia: A survey of head doctors and insurance administrators [77]	J. L. Twigg/2002	Health care reform/Survey	Russia
62	Old Age, Disability, and Survivors [78]	SSPTW: Europe/2002	Old Age/Report	Romania
63	Reforming health insurance in Argentina and Chile [79]	A Barrientos 1, P Lloyd-Sherlock/2000	Reforming health insurance/Survey	Argentina
64	Profile of the Health Services System of Cuba [80]	Pan American health organization/1999	Health Services System/Review	Cuba
65	Health Care Systems in Transition Russian Federation [81]	World Health Organization/1998	Health Services System/Review	Russia
66	Healthcare provision for elderly people in Argentina: The crisis of PAMI [82]	P. LloydSherlock/1997	Health insurance programmes/Original research	Argentina

As you can see, the countries’ insurance plans or health and care financing programmes are described below (Table 3):

**Table 3:** Healthcare financing systems for the elderly

Country	Plan		revenue raising				pooling of funds	purchasing of services
			government budgets	compulsory or voluntary prepaid insurance schemes	OOP^*^	external aid		
Argentina	(contributory state pension scheme)		Government financing in terms of taxes	The contribution rate is a total of 20%	-	-	Social insurance funds (known as obras sociales)	Fixed benefits at around 28% of average salary Personal contribution scheme^***^
	PAMI(El Programa de Atención Médica Integral)		Receive substantial subsidies from the federal government	1% of the monthly salary of all salaried workers, Pension tax 3%, 10% of National Pension Scheme income		Loans from the National Social Security Administration		Primary health care (provided through approved general practitioners) Secondary and tertiary care^****^
	Private health care system^*****^		-	Full premium payment by the older adult	-	-	Private health care system	It covers acute care, outpatient care and doctor’s visits.
Cuba	casas de abuelos		General tax	-	-	-	National Health System	Prevention and continuous monitoring of disabilities, special attention to patients with reduced mental function, and health promotion
	National Health System (SNS) OR national public health system							Free preventive, curative and rehabilitative services
	Private financing		-	-	Small payments from people’s	-	Private financing	Medicines prescribed for outpatients are hearing aids, dental and orthopedic prostheses, and medical equipment such as wheelchairs and crutches.
Romania	The pension and health insurance systems and the system of social welfare services	Public pensions	-	The social insurance premium rate for most employees is 31.3%, of which 20.8% is paid by the employer and 10.5% by the employee.	-	-	Older adult insurance	It responds to the specific needs of older adult Romanians
		Compulsory private pensions						
		Private discretionary pension						
	health insurance		Government funding through taxes	National Health Insurance Fund			National Health Insurance Fund	Receive medical services in outpatient clinics and hospitals that have contracts with health insurance funds.
	National Strategy on Active Ageing, Promotion and Protection of Older adult and the Action Plans 2015–2020’							Employment, education, prevention of the risk of abuse and neglect, is long-term. Care and participation of the older adult in voluntary efforts.
	Romanian Retirement Mutual Aid Associations(CARP)		-	-	-	income from economic activities	CARP	Specialized medical practices in gerontology, dentistry, medical imaging, ophthalmology
	long term care		Local budgets, state budgets	-	-	-	LTC	Care of the older adult at home (nursing homes)_Institutional care (residential care)
			-	-	Private financing	-		
Russia	“social houses” and “homes for older adult.”		Government budget	-	-	-	Insurance funds	Temporary care in social homes and permanent care in nursing homes
	(MHI) mandatory health insurance OR (FOMIF) Russian Federation is Federal Obligatory Medical Insurance			Compulsory insurance premium				General medical services, specialized medical care and the provision of medicine are used.
Thailand	Universal Coverage Scheme (UCS)		income tax	-	14%^**^	-	UHC fund	Outpatient and inpatient care with an emphasis on health promotion and prevention.
	Medical Welfare Scheme (MWS)				-		MWS(Integration with voluntary health card scheme and conversion to UCS)	Providing free inpatient and outpatient services
	long-term care insurance (LTCI)		Government revenue	-	Family trustees are the main source of LTC financing in Thailand		LTCI	(1) residential homes, (2) assisted living settings, (3) long-term care hospitals, (4) nursing homes, and (5) hospice care
	National plan for the older adult		Government funding(UHC)	-	-		Tambon Health Fund	Health-related care services
	(VHVs) Village Health Volunteers		-	-	-	Voluntary contributions	VHVs	Health-related support to the older adult in the community
Ukraine	Semashko model		Government funding	-	42.3٪	-	Semashko model	Providing inpatient and outpatient health care
	Free health services program		National and local budgets funded by taxes	-	-	-	Free health services program	• Emergency care • Polyclinic outpatient care • Inpatient care • Emergency dental care • First aid for the rural population • Specialized nursing homes • Medical care.
	Long-term care in Ukraine						LTC	Medical-social care
Sri Lanka	Agrahara scheme		Government tax revenue and private expenditure	*	-	-	Social health insurance	Inpatient care with more benefits
	Health care system		Government tax revenue and private expenditure	-	-	* Especially after the 2004 tsunami		Providing services for free
	Protection of the Rights of Elders act, No. 9 of 2000		Government tax revenue and private expenditure	-	*	*	National Council of the Older adult	Home care services, provision of assistive devices for the older adult with disabilities, financial assistance for the needy, free legal advice services and support for income-generating activities.
	LTC		Government tax revenue and private expenditure	-	* There are in the private sector such as HelpAge daily center	*	LTC and HelpAge	Provide comprehensive health care

Note’s: *out-of-pocket payments

** Out-of-pocket healthcare costs have decreased from 33% in 2001 to 14% in 2011. Co-pay for each covered item

*** A funded model is based on individual accounts where individuals collect personal contributions (net of administrative costs and disability and survivors insurance) and receive a defined contribution benefit at retirement

**** The program’s core medical services were complemented by a range of social provisions, including emergency food packages, funeral expenses, increased means-tested pensions, and a national network of day care centers

***** Only 8% of Argentine seniors purchase services from private health insurance systems

### Argentina

The Argentine health system is fragmented into public, social security, and private sectors. Older adult coverage is mainly through PAMI, a contributory health fund financed by payroll and pension-related taxes [69, 70, 73, 75, 79, 82]. While primary and hospital care are provided, LTC services are limited and unevenly distributed [73]. [Further details in Appendix]

### Cuba

Cuba has a tax-based universal health system (SNS) with state ownership of facilities and personnel. Older adult care is supported by state pensions and programs such as casas de abuelos [37, 61, 72, 76, 83]. While access is universal, emerging private payments for medicines and devices represent a financing gap [76]. [Further details in Appendix]

### Romania

Romania relies on a three-pillar pension system and compulsory social health insurance, supplemented by local budgets and NGOs for long-term care of the older adult. Mutual aid associations (CARP) provide medical, social and community services. Policies emphasize “active aging,” but financing remains fragmented [26, 45, 56, 78]. [Further details in Appendix]

### Russia

Healthcare is mainly financed through compulsory medical insurance (FOMIF) and government transfers. The older adult rely heavily on government pensions, and nursing homes and care homes provide the necessary care. Insurance coverage is generally broad, but benefit packages lack transparency and LTC remains underfunded [24]. [Further details in Appendix]

### Sri Lanka

Sri Lanka operates a tax-funded health system with free universal services. Additional coverage is offered via Agrahara, a social health insurance scheme for public employees. LTC is mostly family-based, with limited institutional capacity, complemented by NGOs such as HelpAge [33, 34, 41, 71]. [Further details in Appendix]

### Thailand

Thailand’s Universal Coverage Scheme (UCS) was developed and implemented in 2002, with universal access financed through taxation. Vulnerable groups, including the older adult, receive targeted benefits supported by Village Health Volunteers (VHV) and Tambon Health Funds. LTC financing is based on households, with the government also increasing its contribution [25, 30, 36, 44, 49, 51, 57, 63, 74]. [Further details in Appendix]

### Ukraine

Ukraine implements the Semashkov model in the country, which finances public services from taxes and provides long-term care to vulnerable groups in nursing homes and social institutions, but financial resources are limited. Despite the universal right, out-of-pocket payments remain high [52]. [Further details in Appendix]

## Discussion

The study findings showed a diversity of health and long-term care financing systems in developing countries for the older adult. This diversity is not only in the structure of the financing system but is also regulated in terms of the economic roots, politics, financial capacities and demographic structure of each country. These variations can be seen in the models below, as follows:

1. Variations in Health Financing Models

In the seven countries studied, three dominant models of health financing were observed:

The first model is tax-based systems (e.g., Cuba, Ukraine, Sri Lanka) that rely on public revenues and reflect a stronger role for the government.

The second model is the social health insurance (SHI) model (e.g., Argentina, Romania, Russia) that rely on payroll contributions and cover mostly formal workers.

And finally, hybrid systems (e.g., Thailand) that combine public taxation with participatory mechanisms.

The differences in these models depend on financial sustainability, access to services, and equity, especially for the older adult. The differences in the models can be summarized as follows: while universal access may be possible in the first model, this model is financially constrained.

2. Impact of Broader Societal Structures

The demographic structure and economic capacity of countries, depending on the active labor force and the percentage of the older adult population, play a very important role in shaping financing approaches. For example, Romania uses a three-tier pension model that focuses on the active participation of the older adult in economic and social activities, while Sri Lanka and Ukraine, despite the high proportion of the older adult in society, face fragmented long-term care systems and even budget shortages in this area.

3. LTC: A Critical Gap

Comprehensive and sustainable long-term care (LTC) systems remain absent in all studied countries. Family-based LTC (Thailand, Sri Lanka) is under strain due to urbanization and social change, while institutional LTC (Russia, Romania) lacks standardization and adequate funding.

4. Financing Gaps and Equity Concerns

Today, due to rising costs and increasing privatization of essential services, out-of-pocket costs are a barrier to older people accessing services, even in countries such as Cuba and Ukraine that have publicly funded systems. Efforts such as Romania’s Active Ageing Strategy or Thailand’s Tambon Health Funds highlight the importance of local government, intersectoral coordination, and community participation in financing and delivering care for the older adult.

5. Policy Implications

The results of the study showed that there is a need to consider integrated financing approaches, preventive policies for vulnerable groups, and attention to preventive care to link health, social protection, and long-term care. The same results showed that countries facing rapid population aging need a hybrid approach that combines tax funding with participatory insurance and community-based long-term care.

The framework for health financing strategies in countries on aging

The issue of financing for health care for the older adult was addressed in each country. Tax-based financing models (e.g., Cuba, Sri Lanka, Ukraine) considered universal access but faced financial constraints. Social health insurance systems (Argentina, Romania, Russia) provided financing but were not well-suited to environmental challenges. Thailand’s hybrid approach achieved broad coverage but was also dependent on family support for long-term care. Therefore, the effectiveness of the schemes was uncertain. In most countries, financing for the older adult was integrated into public health financing systems (e.g., UCS in Thailand, Semashko in Ukraine). Argentina’s PAMI was a minor exception, operating as a semi-autonomous scheme in a fragmented system. There were no separate financing systems for the older adult, and most governments had designed a hybrid financing system.

Key similarities in the design of older adult care financing programs were the focus on blended financing mechanisms, the pooling of financial risk, and the focus on the family aspect of financing long-term care. Comprehensive, long-term strategies for aging populations were such that few countries explicitly linked financing to broader aging strategies. Romania’s active aging strategy linked pensions, health, and long-term care (LTC), while Thailand developed community-based long-term care through tambon health funds and village health volunteers. Other countries lacked comprehensive intersectoral aging strategies and focused solely on financing mechanisms.

## Conclusion

The review of the countries studied found major challenges in financing health care for older people. Most countries have focused on general schemes rather than specific policies for older people, with Thailand and Romania having specific policies for older people, and neither providing comprehensive long-term care systems. The review of policies highlighted key issues, despite the growing demand due to the ageing population. These include a lack of funding for dedicated long-term care programmes and integrated models. Informal family care remains prevalent but unsustainable.

Policy recommendations for countries include:

- Expanding financing for vulnerable and older adult populations.
- Investing in community-based long-term care models.
- Establishing volunteer groups and training programs for older adult care.
- Integrating health, social, and voluntary care systems.
- Strengthening monitoring systems for health costs and outcomes in older adult care.

Achieving universal health coverage requires health financing reforms based on policy recommendations for countries according to economic contexts and demographic structure, etc. that explicitly address the needs of older people. More research is needed on cost-effective and country-adapted LTC models.

## Electronic supplementary material

Below is the link to the electronic supplementary material.

Supplementary Material 1

## Data Availability

All data generated or analysed during this study are included in this published article [and its supplementary information files].

## Abbreviations

PAMI: Programa de Atencion Medica Integral
HMOs: Health maintenance organization
SNS: Sistema Nacional de Salud
CARP: Romanian Mutual Aid Associations for the Older adult
LTC: Long-term care
NGOs: Non-governmental organizations
FOMIF: Russian Federation is Federal Obligatory Medical Insurance
UCS: Universal Coverage Scheme
NHSO: National Health Security Administration’s
VHVs: Village Health Volunteers
MHI: Mandatory health insurance
MWS: Medical Welfare Scheme
LTCI: Long-term care insurance
UHC: Universal Health Coverage
MOF: Ministry of Finance
MOH: Ministry of Health

## Declarations

### Research limitations

Demographic shifts, varying national contexts and linguistic diversity necessitate cross-national policy analysis. Since countries develop unique programs based on their specific conditions and language frameworks, a comprehensive multilingual examination of policies becomes essential. A more in-depth and tailored critical analysis of the program’s implementation and impact, using established health financing frameworks, was beyond the scope of this descriptive review and warrants separate studies.
