# Supplementary material for: Health financing policies for aging populations: a comparative study of seven countries
Source: BMC Health Serv Res. 2025 Nov 26;25:1535. doi: 10.1186/s12913-025-13648-y (PMC12659251; doi:10.1186/s12913-025-13648-y)
Supplement: Supplementary file 1 — Supplementary Material 1 [file 12913_2025_13648_MOESM1_ESM.docx]

Appendix 1

| **Country** | **Financing Sources** | **Older adult Coverage Schemes** | **LTC (Long-Term Care)** | **Key Challenges** |
| --- | --- | --- | --- | --- |
| **Argentina** | Public sector (taxes), Social health insurance (*obras sociales*), Private insurance | *PAMI* (Programa de Atención Médica Integral), funded by payroll & pension-related taxes | Limited institutional LTC, mostly home-based; private care underdeveloped | Highly fragmented system, inequity in access |
| **Cuba** | General taxation (100%) | *SNS* (Sistema Nacional de Salud) covers entire population; older adult supported by pensions & *casas de abuelos* | State-run older adult day-care centers; universal access but limited resources | Emerging private payments for medicines & devices |
| **Romania** | Social Health Insurance (65%), State/local budgets, Private sources (NGOs, CARP) | Public & mandatory private pensions; *CARP* offers medical, social & community services | Institutional LTC (residential care) plus NGO support; tax & insurance-based | Fragmented financing, sustainability issues |
| **Russia** | Government budgets, Compulsory Medical Insurance (FOMIF), Voluntary insurance | State pensions + universal health coverage under FOMIF | Social homes (temporary) & nursing homes (permanent); both public & private | Benefit package unclear; underfunded LTC |
| **Sri Lanka** | Tax-funded universal health services, *Agrahara* social health insurance, Private & NGO support | Universal access to PHC; *Agrahara* covers public employees | Mostly family-based LTC; limited institutional capacity; NGOs (e.g., HelpAge) play role | Rising OOP payments, weak LTC infrastructure |
| **Thailand** | General taxation, Social health insurance, Private insurance | *UCS* provides near-universal coverage; older adult receive targeted benefits | Community-based LTC; Tambon Health Funds; Village Health Volunteers support home care | Heavy reliance on family financing; decentralization challenges |
| **Ukraine** | State budget (taxes: VAT, corporate, trade, consumption) | Universal access under Semashko model; free healthcare in principle | Nursing homes for older adult, disabled & retired; tax-funded institutional care | High OOP payments; resource shortages; equity concerns |

Appendix 2

Keywords: (aging or aged or adult or older or elderly) and ("health insurance" or "health program" or "health programs" or "health financing" or "healthcare financing" or "health expenditure" or "healthcare expenditure" or "health cost" or "health costs" or "healthcare cost" or "healthcare costs" or "financial support") and (Romania or Ukraine or Cuba or Russia or Thailand or Argentina or China or Sri Lanka)

Search strategy

Ovid:

1 (aging or aged or adult or older or elderly).ti. or (aging or aged or adult or older or elderly).ab. or (aging or aged or adult or older or elderly).kw. =506254

2 ("health insurance" or "health program" or "health programs" or "health financing" or "healthcare financing" or "health expenditure" or "healthcare expenditure" or "health cost" or "health costs" or "healthcare cost" or "healthcare costs" or "financial support").ti. or ("health insurance" or "health program" or "health programs" or "health financing" or "healthcare financing" or "health expenditure" or "healthcare expenditure" or "health cost" or "health costs" or "healthcare cost" or "healthcare costs" or "financial support").ab. or ("health insurance" or "health program" or "health programs" or "health financing" or "healthcare financing" or "health expenditure" or "healthcare expenditure" or "health cost" or "health costs" or "healthcare cost" or "healthcare costs" or "financial support").kw.=18359

3 (Romania or Ukraine or Cuba or Russia or Thailand or China or Argentina).ti. or (Romania or Ukraine or Cuba or Russia or Thailand or China or Argentina).ab. or (Romania or Ukraine or Cuba or Russia or Thailand or China or Argentina).kw.=41645

4 1 and 2 and 3=68

PubMed:

((((((aging[Title/Abstract]) OR (aged[Title/Abstract])) OR (adult[Title/Abstract])) OR (older[Title/Abstract])) OR (elderly[Title/Abstract]) AND (english[Filter])) AND ((((((((((((((((("health insurance"[Title/Abstract]) OR ("health program"[Title/Abstract])) OR ("health programs"[Title/Abstract])) OR ("health financing"[Title/Abstract])) OR ("healthcare financing"[Title/Abstract])) OR ("health expenditure"[Title/Abstract])) OR ("healthcare expenditure"[Title/Abstract]))) OR ("health cost"[Title/Abstract])) OR ("health costs"[Title/Abstract])) OR ("healthcare cost"[Title/Abstract])) OR ("healthcare costs"[Title/Abstract])) OR ("financial support"[Title/Abstract]))) OR ("health plan"[Title/Abstract])) OR ("health plans"[Title/Abstract])) AND (english[Filter]))) AND (((((((Romania[Title/Abstract]) OR (Ukraine[Title/Abstract])) OR (Cuba[Title/Abstract])) OR (Russia[Title/Abstract])) OR (Thailand[Title/Abstract])) OR (China[Title/Abstract])) OR (Argentina[Title/Abstract]) AND (english[Filter])) English (("aging"[Title/Abstract] OR "aged"[Title/Abstract] OR "adult"[Title/Abstract] OR "older"[Title/Abstract] OR "elderly"[Title/Abstract]) AND "english"[Language] AND (("health insurance"[Title/Abstract] OR "health program"[Title/Abstract] OR "health programs"[Title/Abstract] OR "health financing"[Title/Abstract] OR "healthcare financing"[Title/Abstract] OR "health expenditure"[Title/Abstract] OR "healthcare expenditure"[Title/Abstract] OR "health cost"[Title/Abstract] OR "health costs"[Title/Abstract] OR "healthcare cost"[Title/Abstract] OR "healthcare costs"[Title/Abstract] OR "financial support"[Title/Abstract] OR "health plan"[Title/Abstract] OR "health plans"[Title/Abstract]) AND "english"[Language]) AND (("Romania"[Title/Abstract] OR "Ukraine"[Title/Abstract] OR "Cuba"[Title/Abstract] OR "Russia"[Title/Abstract] OR "Thailand"[Title/Abstract] OR "China"[Title/Abstract] OR "Argentina"[Title/Abstract]) AND "english"[Language])) AND (english[Filter]) 583 08:03:02

Web of Science:

Scopus:

74: ( TITLE-ABS-KEY ( aging ) OR TITLE-ABS-KEY ( aged ) OR TITLE-ABS-KEY ( adult ) OR TITLE-ABS-KEY ( older ) OR TITLE-ABS-KEY ( elderly ) )

12,186,174 document results

75: ( TITLE-ABS-KEY ( "health insurance" ) OR TITLE-ABS-KEY ( "health program" ) OR TITLE-ABS-KEY ( "health programs" ) OR TITLE-ABS-KEY ( "health financing" ) OR TITLE-ABS-KEY ( "healthcare financing" ) OR TITLE-ABS-KEY ( "health expenditure" ) OR TITLE-ABS-KEY ( "healthcare expenditure" ) OR TITLE-ABS-KEY ( "health cost" ) OR TITLE-ABS-KEY ( "health costs" ) OR TITLE-ABS-KEY ( "healthcare cost" ) OR TITLE-ABS-KEY ( "healthcare costs" ) OR TITLE-ABS-KEY ( "financial support" ) ) ...

380,333 document results

76: ( TITLE-ABS-KEY ( romania ) OR TITLE-ABS-KEY ( ukraine ) OR TITLE-ABS-KEY ( cuba ) OR TITLE-ABS-KEY ( russia ) OR TITLE-ABS-KEY ( thailand ) OR TITLE-ABS-KEY ( china ) OR TITLE-ABS-KEY ( argentina ) )

1,539,207 document results

77: ( ( TITLE-ABS-KEY ( aging ) OR TITLE-ABS-KEY ( aged ) OR TITLE-ABS-KEY ( adult ) OR TITLE-ABS-KEY ( older ) OR TITLE-ABS-KEY ( elderly ) ) ) AND ( ( TITLE-ABS-KEY ( "health insurance" ) OR TITLE-ABS-KEY ( "health program" ) OR TITLE-ABS-KEY ( "health programs" ) OR TITLE-ABS-KEY ( "health financing" ) OR TITLE-ABS-KEY ( "healthcare financing" ) OR TITLE-ABS-KEY ( "health expenditure" ) OR TITLE-ABS-KEY ( "healthcare expenditure" ) OR TITLE-ABS-KEY ( "health cost" ) OR TITLE-ABS-KEY ( "health costs" ) OR TITLE-ABS-KEY ( "healthcare cost" ) OR TITLE-ABS-KEY ( "healthcare costs" ) OR TITLE-ABS-KEY ( "financial support" ) ) ) AND ( ( TITLE-ABS-KEY ( romania ) OR TITLE-ABS-KEY ( ukraine ) OR TITLE-ABS-KEY ( cuba ) OR TITLE-ABS-KEY ( russia ) OR TITLE-ABS-KEY ( thailand ) OR TITLE-ABS-KEY ( china ) OR TITLE-ABS-KEY ( argentina ) ) ) ...

4,227 document results

78: ( ( TITLE-ABS-KEY ( aging ) OR TITLE-ABS-KEY ( aged ) OR TITLE-ABS-KEY ( adult ) OR TITLE-ABS-KEY ( older ) OR TITLE-ABS-KEY ( elderly ) ) ) AND ( ( TITLE-ABS-KEY ( "health insurance" ) OR TITLE-ABS-KEY ( "health program" ) OR TITLE-ABS-KEY ( "health programs" ) OR TITLE-ABS-KEY ( "health financing" ) OR TITLE-ABS-KEY ( "healthcare financing" ) OR TITLE-ABS-KEY ( "health expenditure" ) OR TITLE-ABS-KEY ( "healthcare expenditure" ) OR TITLE-ABS-KEY ( "health cost" ) OR TITLE-ABS-KEY ( "health costs" ) OR TITLE-ABS-KEY ( "healthcare cost" ) OR TITLE-ABS-KEY ( "healthcare costs" ) OR TITLE-ABS-KEY ( "financial support" ) ) ) AND ( ( TITLE-ABS-KEY ( romania ) OR TITLE-ABS-KEY ( ukraine ) OR TITLE-ABS-KEY ( cuba ) OR TITLE-ABS-KEY ( russia ) OR TITLE-ABS-KEY ( thailand ) OR TITLE-ABS-KEY ( china ) OR TITLE-ABS-KEY ( argentina ) ) ) AND ( LIMIT-TO ( LANGUAGE , "English" ) ) ...

3,890 document results

ProQuest:

TI,AB,IF(aging or aged or adult or older or elderly) AND TI,AB,IF("health insurance" or "health program" or "health programs" or "health financing" or "healthcare financing" or "health expenditure" or "healthcare expenditure" or "health cost" or "health costs" or "healthcare cost" or "healthcare costs" or "financial support") AND TI,AB,IF(romania or ukraine or cuba or russia or thailand or china or argentina)

522 document results
